# Evaluation of protein biomarkers of prostate cancer aggressiveness

**DOI:** 10.1186/1471-2407-14-244

**Published:** 2014-04-05

**Authors:** Anthony E Rizzardi, Nikolaus K Rosener, Joseph S Koopmeiners, Rachel Isaksson Vogel, Gregory J Metzger, Colleen L Forster, Lauren O Marston, Jessica R Tiffany, James B McCarthy, Eva A Turley, Christopher A Warlick, Jonathan C Henriksen, Stephen C Schmechel

**Affiliations:** 1 Department of Pathology, University of Washington, Mailcode 359791, 908 Jefferson St, Seattle, WA 98104, USA; 2 Department of Laboratory Medicine and Pathology and Masonic Cancer Center, University of Minnesota, Minneapolis, MN, USA; 3 Biostatistics and Bioinformatics Core, Masonic Cancer Center, University of Minnesota, Minneapolis, MN, USA; 4 Division of Biostatistics, School of Public Health, University of Minnesota, Minneapolis, MN, USA; 5 Department of Radiology, University of Minnesota, Minneapolis, MN, USA; 6 BioNet, Academic Health Center, University of Minnesota, Minneapolis, MN, USA; 7 Department of Biochemistry, London Health Sciences Center, University of Western Ontario, London, Ontario, Canada; 8 Department of Oncology, London Health Sciences Center, University of Western Ontario, London, Ontario, Canada; 9 Department of Urology, University of Minnesota, Minneapolis, MN, USA

**Keywords:** Prostate cancer, Aggressiveness, Biomarker, Signature

## Abstract

**Background:**

Prognostic multibiomarker signatures in prostate cancer (PCa) may improve patient management and provide a bridge for developing novel therapeutics and imaging methods. Our objective was to evaluate the association between expression of 33 candidate protein biomarkers and time to biochemical failure (BF) after prostatectomy.

**Methods:**

PCa tissue microarrays were constructed representing 160 patients for whom clinicopathologic features and follow-up data after surgery were available. Immunohistochemistry for each of 33 proteins was quantified using automated digital pathology techniques. Relationships between clinicopathologic features, staining intensity, and time to BF were assessed. Predictive modeling using multiple imputed datasets was performed to identify the top biomarker candidates.

**Results:**

In univariate analyses, lymph node positivity, surgical margin positivity, non-localized tumor, age at prostatectomy, and biomarkers CCND1, HMMR, IGF1, MKI67, SIAH2, and SMAD4 in malignant epithelium were significantly associated with time to BF. HMMR, IGF1, and SMAD4 remained significantly associated with BF after adjusting for clinicopathologic features while additional associations were observed for HOXC6 and MAP4K4 following adjustment. In multibiomarker predictive models, 3 proteins including HMMR, SIAH2, and SMAD4 were consistently represented among the top 2, 3, 4, and 5 most predictive biomarkers, and a signature comprised of these proteins best predicted BF at 3 and 5 years.

**Conclusions:**

This study provides rationale for investigation of HMMR, HOXC6, IGF1, MAP4K4, SIAH2, and SMAD4 as biomarkers of PCa aggressiveness in larger cohorts.

## Background

Although prostate cancer (PCa) is the most commonly diagnosed non-cutaneous cancer of men in the United States [1], few prognostic biomarkers are available for routine clinical use. Serum PSA biochemical failure (BF) versus non-failure is a well-established binary outcome variable. Since clinical treatment failure (systemic progression and/or local recurrence) is essentially always preceded by BF (by a median of 8 years) [2-5], PSA non-failure has high negative predictive value (NPV) for poor clinical outcome after prostatectomy. When identifying and validating new potential tissue based biomarkers for the purpose of attempting to improve prognostication after prostatectomy, the greatest clinical risk would be to mischaracterize biologically aggressive disease as “non-aggressive,” since that would lead to erroneous underestimation of the malignant potential of the disease. For this reason, we seek tissue based biomarkers that maximally correlate with PSA failure.

Multibiomarker signatures and gene associations have been implicated in PCa progression but few markers emerge among multiple independent studies as potentially prognostic, outperform established clinicopathologic parameters in predicting BF following prostatectomy, or have become routinely used in clinical labs [6-18]. Current prognostic indicators in PCa include preoperative PSA, tumor stage, and grade [19]. Prognostic molecular biomarkers may identify pathways that can be exploited for therapy. For example, the TMPRSS2/ERG gene fusion was identified as a frequent chromosomal rearrangement present in a subgroup of prostate tumors that leads to ERG transcription factor overexpression [20]. Subsequent studies have demonstrated that inhibiting this pathway by delivery of liposomal nanovectors carrying siRNA specific for the TMPRSS2/ERG fusion transcript provides therapeutic advantage in a murine PCa model [21]. Additionally, multibiomarker signatures can be spatially quantified to aid in the development of novel imaging methods to assess PCa aggressiveness preoperatively *in vivo* through co-registration of postoperative pathology data with preoperative imaging data [22].

Numerous studies have described biomarkers of aggressive biologic behavior in PCa such as those correlated with aberrant hyaluronan (HA) processing [8,11,12], neuroendocrine phenotype [14,23], increased tumor angiogenesis [24], and poor prognosis [7,25]. Other groups have developed multi-biomarker signatures of PCa aggressiveness using gene expression profiling as a method for biomarker discovery; these studies have surprisingly little overlap between gene sets [6,9,10,13,15-18]. In this study, we took a combined approach of a) non-biased, cross-study examination of gene expression profiling data and b) a candidate gene approach (gene products that appear to be associated with PCa outcome in prior publications), to identify 33 potential aggressiveness biomarkers. We evaluated the association of these biomarkers with BF using immunohistochemistry and automated digital pathology techniques on tissue microarrays (TMAs) representing PCa tissue from 160 prostatectomy specimens.

## Methods

### Selection of candidate proteins

We identified 33 genes for study at the protein level by immunohistochemistry. Eleven genes were selected based on our own cross-study analysis of three publicly available PCa gene expression profiling datasets as described in Additional files 1, 2, 3, 4, 5, 6, 7, 8, 9, 10, 11, 12 (ACPP, ADAM9, ALDH1A2, CASR, CCPG1, GADD45B, HOXC6, IGF1, IQCK, PAGE4, PLIN2). Four genes were included based on their occurrence in multiple published gene signatures of PCa aggressiveness (CHMP1A, EI24, MAP4K4, MKI67) [6,9,16,17]. Additional candidates were included based on PCa literature review and included: 6 genes associated with HA processing in PCa (HA, HAS2, HMMR, HYAL1, CD44, CD44v6) [8,11,12]; 4 genes implicated in poor prognosis of PCa (CCND1, PTEN, SMAD4, SPP1) [7]; 3 genes characteristic of the neuroendocrine prostate tumor phenotype (CHGA, ENO2, SYP) [23]; 3 genes associated with development of neuroendocrine prostate tumors (HES6, SIAH2, SOX9) [14]; 1 marker of tumor angiogenesis (CD34) [24]; and 1 tumor suppressor gene associated with poor progression (TP53) [25].

### Clinical cohort and TMA construction

Archival formalin-fixed paraffin-embedded tissues from patients with Gleason score 6, 7, 8, and 9 prostate acinar/conventional adenocarcinomas that underwent radical prostatectomy at the University of Minnesota Medical Center Fairview from 1999 to 2008 were retrospectively collected after approval from the University of Minnesota Institutional Review Board. Patients with tumors predominantly containing Gleason pattern 5 (5 + 4 and 5 + 5 morphology) were excluded. Our study focused on biomarker expression in tumor cells comprising primary Gleason patterns 3 and 4. Molecular evidence suggests that tumor of Gleason pattern 5 morphology is biologically distinct from tumor of Gleason patterns 3 and 4 [26,27]. Further, there is epidemiological evidence that Gleason pattern 4 represents a pattern intermediate between lower risk (pattern 3) and much higher risk (pattern 5) prostate cancer. For these reasons, we believe that tumor cells of pattern 3 (3 + 3) and pattern 4 (4 + 4, and the pattern 4 component of 3 + 4, 4 + 3, and 4 + 5 tumors) are of most interest in prognostic biomarker studies. Demographic and clinical parameters were abstracted from preexisting pathology reports and electronic medical records. Representative PCa areas were identified on hematoxylin and eosin-stained sections for each case. TMAs consisting of quadruplicate 1.0 mm core samples were constructed with a manual tissue arrayer (MTA-1, Beecher, Sun Prairie, WI).

### Immunohistochemistry

Unstained, 4 μm-thick sections were deparaffinized and rehydrated using standard methods. Table 1 contains detailed information on sources, dilutions, antigen retrieval, and detection methods for each antibody. Antibodies were optimized with positive and negative control tissues and patterns of expression were demonstrated to be highly similar to those seen in previous publications (Additional file 13: Table S12). Most antibodies required standard antigen retrieval; slides were incubated in 6.0 pH buffer (Reveal Decloaker; Biocare Medical, Concord, CA) in a steamer for 30 min at 95-98°C, followed by a 20 min cool down period. Slides were rinsed in 1 × Tris-buffered saline/0.1% Tween-20 (TBST; pH 7.4). Subsequent steps were automated using a robotic staining platform (Nemesis; Biocare), except MKI67 and TP53 immunohistochemistry which were performed on a Ventana platform using the manufacturer’s specifications (Ventana Medical Systems, Tucson, AZ). Endogenous peroxidase activity was quenched with 3% hydrogen peroxide solution (Peroxidazed; Biocare) for 10 min followed by rinsing. Serum-free blocking solution (Background Sniper; Biocare) was applied for 10 min. Blocking solution was removed and slides were incubated with primary antibody diluted in 10% blocking solution/90% TBST. Primary antibody incubations were performed according to Table 1. Most detection was performed with the Novocastra Novolink Polymer Kit (Leica Microsystems, Buffalo Grove, IL). Detection for HAS2 was performed with the VECTASTAIN Elite ABC Kit (Vector, Burlingame, CA). Histochemical detection of HA was performed using biotinylated HA binding protein (bHABP) and only required the tertiary reagent of the VECTASTAIN Elite ABC Kit (Vector). To demonstrate specificity for bHABP, additional sections were pre-treated in 0.1 M sodium acetate buffer (pH 5.0)/1% hyaluronidase (Sigma) for 45 min at 37°C (data not shown). Detection for HMMR was performed with a streptavidin-horseradish peroxidase (HRP) kit (Covance, Princeton, NJ). Final detection steps were completed using 3,3-diaminobenzidine (DAB; Covance). Slides were incubated for 5 min followed by rinsing, counterstaining (CAT Hematoxylin; Biocare), dehydrating, and coverslipping. A second independent run of immunohistochemistry and image analysis was performed for a subset of biomarkers to examine reproducibility.

**Table 1:** Antibodies and conditions used for immunohistochemistry assays

**Antibody**	**Specificity**	**Clone**	**Dilution**	**Antigen retrieval**	**Incubation**	**Detection**	**Source**	**Cat #**
ACPP ACPP	Polyclonal (rabbit)	-	1:2500	Steamer/Citrate pH 6	1 h	Novocastra Novolink Polymer	Sigma-Aldrich	HPA004335
ADAM9	Polyclonal (rabbit)	-	1:100	Steamer/Citrate pH 6	1 h	Novocastra Novolink Polymer	Sigma-Aldrich	HPA004000
ALDH1A2	Polyclonal (rabbit)	-	1:1000	Steamer/Citrate pH 6	1 h	Novocastra Novolink Polymer	Sigma-Aldrich	HPA010022
CASR	Polyclonal (rabbit)	-	1:200	Steamer/Citrate pH 6	1 h	Novocastra Novolink Polymer	Abcam	ab18200
CCND1	Monoclonal (mouse)	DCS-6	1:50	Decloaker/Citrate pH 6	1 h	Novocastra Novolink Polymer	Dako	M7155
CCPG1	Polyclonal (rabbit)	-	1:400	Steamer/Citrate pH 6	1 h	Novocastra Novolink Polymer	Sigma-Aldrich	HPA026861
CD34	Monoclonal (mouse)	QBEnd/10	1:4000	Steamer/Citrate pH 6	1 h	Novocastra Novolink Polymer	Neomarkers	MS-363-P
CD44	Monoclonal (mouse)	SP37	1:400	Steamer/Citrate pH 6	O/N	Novocastra Novolink Polymer	Dako	M708201-2
CD44v6	Monoclonal (mouse)	2 F10	1:1000	Steamer/Citrate pH 6	1 h	Novocastra Novolink Polymer	R&D Systems	BBA13
CHGA	Monoclonal (mouse)	LK2H10 + PHE5	1:800	Steamer/Citrate pH 6	1 h	Novocastra Novolink Polymer	Biocare Medical	CM010B
CHMP1A	Polyclonal (rabbit)	-	1:100	Steamer/Citrate pH 6	1 h	Novocastra Novolink Polymer	Sigma-Aldrich	HPA006776
EI24	Polyclonal (rabbit)	-	1:8000	Steamer/Citrate pH 6	1 h	Novocastra Novolink Polymer	Sigma-Aldrich	SAB1100756
ENO2	Monoclonal (mouse)	5E2	1:800	No AR	1 h	Novocastra Novolink Polymer	Leica	NCL-L-NSE2
GADD45B	Polyclonal (rabbit)	-	1:25	Steamer/Citrate pH 6	1 h	Novocastra Novolink Polymer	Sigma-Aldrich	HPA029816
HABP^a^	NA (bovine)	-	1:400	No AR	O/N	VECTASTAIN Elite ABC^b^	Calbiochem	385911
HAS2	Polyclonal (goat)	-	1:1000	Steamer/Citrate pH 6	1 h	VECTASTAIN Elite ABC	Santa Cruz	sc-34067
HES6	Polyclonal (rabbit)	-	1:1000	Steamer/Citrate pH 6	O/N	Novocastra Novolink Polymer	Abcam	ab66461
HMMR	Monoclonal (mouse)	6B7D8	1:900	Steamer/Citrate pH 6	1 h	Covance Ultra Streptavidin-HRP	ProMab	NA
HOXC6	Polyclonal (rabbit)	-	1:200	Steamer/Citrate pH 6	1 h	Novocastra Novolink Polymer	Santa Cruz	sc-66925
HYAL1	Polyclonal (rabbit)	-	1:100	Steamer/Citrate pH 6	O/N	Novocastra Novolink Polymer	Sigma-Aldrich	HPA002112
IGF1	Polyclonal (rabbit)	-	1:400	Steamer/Citrate pH 6	1 h	Novocastra Novolink Polymer	Millipore	07-1411
IQCK	Polyclonal (rabbit)	-	1:70	Steamer/Citrate pH 6	1 h	Novocastra Novolink Polymer	Sigma-Aldrich	HPA026792
MAP4K4	Polyclonal (rabbit)	-	1:100	Steamer/Citrate pH 6	1 h	Novocastra Novolink Polymer	Sigma-Aldrich	HPA008476
MKI67	Monoclonal (mouse)	MM1	Predilute	Decloaker/CCS1	32 min	Ventana ultraView Universal	Leica	ORG-8772
PAGE4	Polyclonal (rabbit)	-	1:2000	Steamer/Citrate pH 6	1 h	Novocastra Novolink Polymer	Sigma-Aldrich	HPA023880
PLIN2	Polyclonal (rabbit)	-	1:100	Steamer/Citrate pH 6	1 h	Novocastra Novolink Polymer	Abbiotec	251533
PTEN	Polyclonal (rabbit)	-	1:200	Steamer/Citrate pH 6	1 h	Novocastra Novolink Polymer	Invitrogen	18-0256
SIAH2	Monoclonal (mouse)	24E6H3	1:50	Steamer/Citrate pH 6	1 h	Novocastra Novolink Polymer	Novus	NB110-88113
SMAD4	Monoclonal (rabbit)	EP618Y	1:100	Steamer/Citrate pH 6	1 h	Novocastra Novolink Polymer	Millipore	04-1033
SOX9	Polyclonal (rabbit)	-	1:200	Steamer/Citrate pH 6	1 h	Novocastra Novolink Polymer	Sigma-Aldrich	HPA001758
SPP1	Monoclonal (rabbit)	EPR3688	1:50	Steamer/Citrate pH 6	1 h	Novocastra Novolink Polymer	Epitomics	2671-1
SYP	Monoclonal (mouse)	27G12	1:400	Steamer/Citrate pH 6	1 h	Novocastra Novolink Polymer	Leica	NCL-SYNAP-299
TP53	Monoclonal (mouse)	DO-7	1:40000	Steamer/Citrate pH 6	1 h	Novocastra Novolink Polymer	Dako	M7001
	Monoclonal (mouse)	Bp53-11	Predilute	Decloaker/CCS1	16 min	Ventana ultraView Universal	Ventana	760-2542

CCS1, Cell conditioning solution 1 (Ventana).

^a^Biotinylated conjugate.

^b^Tertiary reagent only.

### Slide digitization, annotation, and immunohistochemical quantification

TMA slides were scanned at 40 × magnification (0.0625 μm^2^/pixel) using a whole slide scanner (ScanScope CS or ScanScope XL; Aperio ePathology, Leica Biosystems, Vista, CA) and preprocessed using the Genie Histology Pattern Recognition software suite (Aperio) to segment tissues into three user-defined Image Classes (tumor, stroma, glass) as previously reported [28]. For markers with predominantly nuclear localization (CCND1, MKI67, SIAH2, and TP53), nuclear DAB staining was quantified within malignant epithelium using the Nuclear algorithm (Aperio). For the microvascular marker (CD34), DAB staining was quantified within whole tumor areas using the Color Deconvolution algorithm (Aperio) for standard area quantification as well as the Microvessel algorithm (Aperio) for quantification of alternative metrics that may be prognostic (average vessel area, average vessel perimeter, average lumen area, average vascular area, and microvessel density). For the remaining markers, DAB staining was quantified within malignant epithelium using the Color Deconvolution algorithm (Aperio), except for HA quantified in tumor-associated stroma and EI24 quantified in both tumor-associated stroma and malignant epithelium (individually). Data obtained using standard Color Deconvolution (Aperio) were summarized by a continuous variable metric that incorporates both the staining strength (measured as average optical density [OD] units since OD is linearly related to amount of DAB staining [29]) and the percentage of positive pixels in malignant epithelium or tumor-associated stromal areas (AvgOD*%Pos). This metric accounts for differences in staining intensities as well as the proportion of positive staining tumor and was previously found to be highly correlated with visual pathologist scoring [28]. Similarly, data obtained using the Nuclear algorithm (Aperio) were summarized as the average staining intensity within nuclei of malignant epithelium (AvgNuclearOD) multiplied by the percentage of positive nuclei in malignant epithelium (AvgNuclearOD*%PosNuclei).

### Statistical analysis

The primary statistical analysis focused on the association between biomarkers and BF. Time to BF was calculated from date of prostatectomy to the date of known BF, defined as the date of a PSA value ≥0.2 ng/mL (taken at least 6 weeks after surgery) and confirmed by a second PSA value >0.2 ng/mL [2]. Time to BF was censored at the last date of contact for subjects who did not experience BF. Patients with only a single post-operative PSA value were excluded. Clinicopathologic features used for statistical analysis included pre-operative PSA (continuous), age at prostatectomy (continuous), primary Gleason pattern (3: 3 + 3 and 3 + 4; or 4: 4 + 3, 4 + 4, and 4 + 5), and Non-Localized Tumor Indicator (yes or no) which was defined by tumor that extended beyond the prostate (pathologic stage pT3), involved lymph nodes (pN1), and/or had positive surgical margin (s) (pR1). Clinicopathologic features and biomarker staining data (averaged across spots by biomarker for each patient) were evaluated for their association with BF using Cox proportional hazards regression. Associations were summarized with the hazard ratio (HR) (per 1 standard deviation difference in biomarker measurement) and 95% confidence interval. P-values of ≤0.05 were considered statistically significant.

In addition, we developed a multibiomarker predictive model for time to BF using a subset of the biomarkers considered in our primary analysis. In order to account for missing biomarker values, ten imputed datasets were generated by chained equations [30] using the ‘mice’ package in R [31]. For each imputed dataset, predictive models were developed for time to BF using the best 2, 3, 4, and 5 biomarkers with variable selection completed using the Lasso [32]. Data were summarized by counting the number of times each biomarker appeared in the top 2, 3, 4 and 5 biomarkers across the ten imputed datasets. From this list, we identified the top 3 biomarkers. The classification accuracy of our multibiomarker predictive model was estimated by calculating the area under the survival ROC curve (AUC) [33] for each imputed dataset and averaging the estimates over the ten imputed datasets. A cross-validation procedure was used to adjust for overfitting and 95% confidence intervals were completed using the bootstrap.

## Results

### Immunohistochemical analysis of PCa related proteins

**Figure 1:**
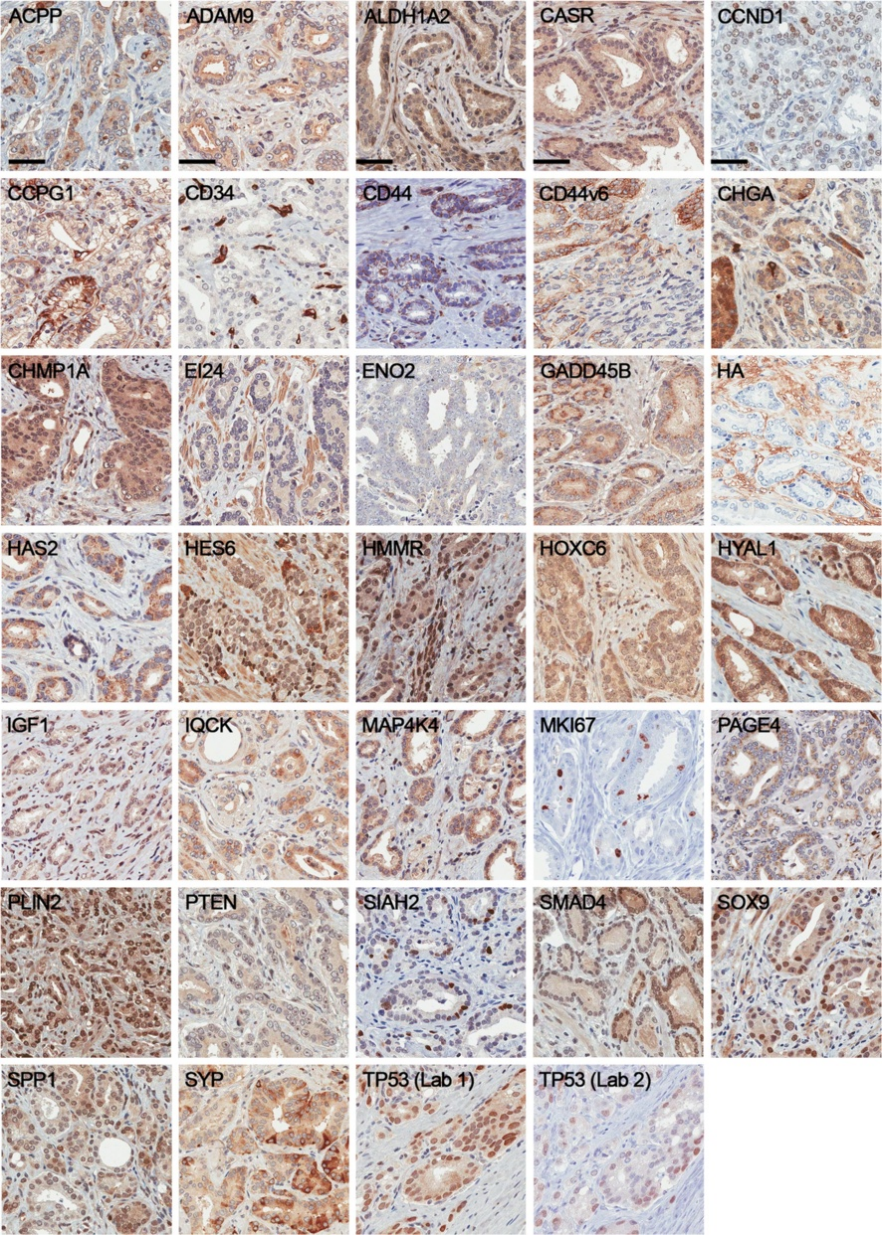
**Immunohistochemistry of candidate biomarkers in prostate cancer.** Representative immunohistochemical staining of ACPP, ADAM9, ALDH1A2, CASR, CCND1, CCPG1, CD34, CD44, CD44v6, CHGA, CHMP1A, EI24, ENO2, GADD45B, HA, HAS2, HES6, HMMR, HOXC6, HYAL1, IGF1, IQCK, MAP4K4, MKI67, PAGE4, PLIN2, PTEN, SIAH2, SMAD4, SOX9, SPP1, SYP, and TP53 from prostate cancer tissue microarrays. Scale bar represents 50 μm.

**Figure 2:**
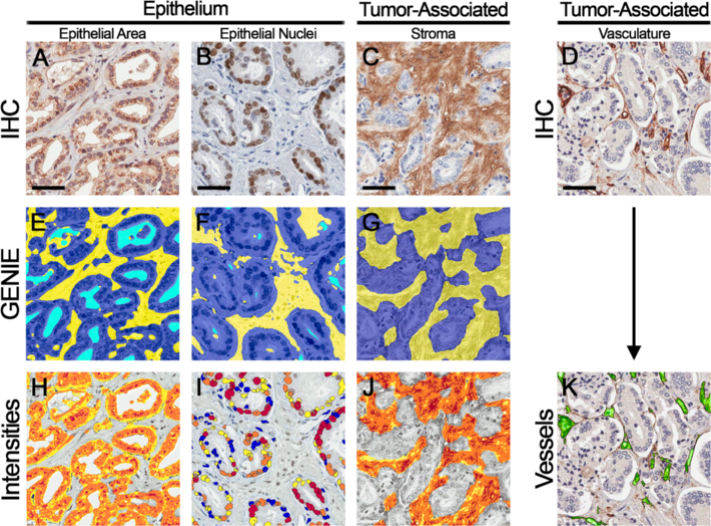
**Image analysis workflow for immunohistochemical staining quantification. (A-D)** Prostate cancer tissue microarrays were stained by immunohistochemistry. The method of image analysis performed independently for each marker was dependent on the staining pattern: default malignant epithelial area **(A)**, malignant epithelial nuclei **(B)**, tumor-associated stromal area **(C)**, or tumor-associated vasculature **(D). (E-G)** Genie Histology Pattern Recognition software (Aperio) subclassified tumor areas into malignant epithelium (dark blue), stroma (yellow), and glass (light blue). **(H)** Markers with heterogeneous positivity were evaluated by Color Deconvolution (Aperio) to quantify staining within malignant epithelial areas. **(I)** Markers with predominantly nuclear localization (CCND1, MKI67, SIAH2, and TP53) were evaluated by the Nuclear algorithm (Aperio) to quantify staining within nuclei of malignant epithelium. **(J)** Markers with significant stromal positivity (EI24 and HA) were evaluated by Color Deconvolution (Aperio) to quantify staining within tumor-associated stroma. **(K)** The microvascular marker (CD34) was evaluated by the Microvessel algorithm (Aperio) to quantify additional metrics including average vessel area, average vessel perimeter, average lumen area, average vascular area, and microvessel density. Scale bars represent 50 μm.

A select group of 33 proteins implicated in advanced PCa or disease progression was evaluated by immunohistochemistry (Table 1) on our PCa cohort TMAs. Immunohistochemical staining patterns were verified using normal and tumor control tissues (described in Additional file 13: Table S12; representative tissues shown in Additional file 14: Figure S1). PCa tissues exhibited positive staining for each of the 33 proteins as illustrated in Figure 1. The image analysis workflow for immunohistochemical staining quantification is displayed in Figure 2.

Additional studies were performed on 8 of the 33 biomarkers (24%) (CCND1, CD44s, CD44v6, HA, HAS2, HMMR, HYAL1, and SMAD4) to investigate the reproducibility of IHC and image analysis. The number of cases with available data varied for each biomarker due to tissue core loss (technical reasons) or exclusion (quality control). For each of the biomarkers, 71.2-84.7% of subjects had evaluable IHC staining on tissue samples for both runs, 0.6-11.8% were missing tissue for one of the two runs, and 8.8-11.8% were missing tissue for both runs (Additional file 15: Table S13). These data losses are within the expected missing data range of 6-30% for TMA-based methodologies [34]. The correlation coefficient for subjects with two independent evaluable marker values ranged from 0.54-0.81 (Additional file 15: Table S13).

### Clinicopathologic features of PCa patients and BF

**Figure 3:**
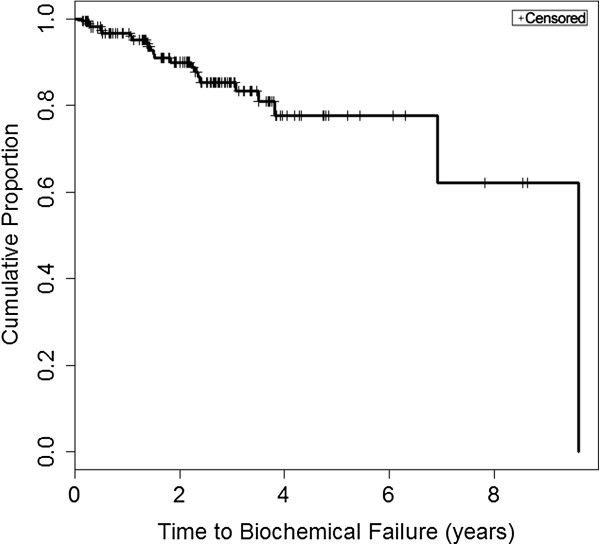
Kaplan-Meier curve demonstrating the time to biochemical failure for the sample population.

A total of 160 subjects met our eligibility criteria and had suitable tissue available for analysis for at least one biomarker. The mean age at the time of prostatectomy was 60.8 years and the average pre-operative PSA was 7.2 ng/mL. A Kaplan-Meier curve for time to BF can be found in Figure 3. Out of 160 patients, 22 experienced BF during follow-up with a median time to BF of 9.6 years (95% CI: 6.9-Inf). The median follow-up among non-failures was 2.3 years (range 50–3156 days). Tumor characteristics are summarized in Table 2. Combined Gleason score, pathologic stage, lymph node involvement, surgical margin involvement, Non-Localized Tumor Indicator, and increased age at prostatectomy were significantly associated with time to BF (Table 2). Preoperative PSA and primary Gleason pattern were not found to be statistically significant; however, multivariate analyses were adjusted for all clinicopathologic features including preoperative PSA and primary Gleason pattern based on established prognostic factors for BF [19].

**Table 2:** Association of clinicopathologic features with time to biochemical failure using univariate Cox regression models

**Variable**	**N**	**%**	**HR (95% CI)**	**P-value**
Total population	160			
Combined Gleason score				0.034
3 + 3	50	31.3	0.09 (0.02-0.51)	
3 + 4	63	39.4	0.17 (0.03-0.78)	
4 + 3	34	21.3	0.17 (0.03-0.92)	
4 + 4	8	5.0	0.11 (0.01-1.23)	
4 + 5	5	3.1	1.00	
Primary Gleason pattern				0.347
3	113	70.6	0.65 (0.27-1.58)	
4	47	29.4	1.00	
Pathologic stage				0.013
Extraprostatic extension (pT3+)	29	18.1	3.13 (1.28-7.69)	
Prostate-limited (pT2)	131	81.9	1.00	
Lymph node involvement				0.018
Yes (pN1)	5	3.1	4.46 (1.29-15.37)	
No (pN0)	155	96.9	1.00	
Surgical margin involvement				0.022
Yes (pR1)	53	33.1	2.82 (1.17-6.84)	
No (pR0)	107	66.9	1.00	
Non-Localized tumor Indicator*				0.005
Yes	67	41.9	4.21 (1.54-11.50)	
No	93	58.1	1.00	
	**N**	**Mean (SD)**	**HR (95% CI)**	**P-value**
Age (years)	160	60.8 (6.8)	1.08 (1.01-1.16)	0.035
Pre-operative PSA (ng/mL)	148	7.2 (5.3)	1.03 (0.98-1.09)	0.238

*Non-Localized Tumor Indicator is a summary metric of tumor confined to prostate. ‘Yes’ indicates tumor growth beyond the prostate (pathologic stage pT3), involved lymph nodes (pN1), and/or positive surgical margins (pR1).

### Prognostic significance of proteins implicated in aggressive prostate cancer

Malignant epithelial staining of CCND1 (nuclear; p = 0.042), HMMR (p = 0.005), IGF1 (p = 0.039), MKI67 (nuclear; p = 0.026), SIAH2 (nuclear; p = 0.016), and SMAD4 (p = 0.010) were significantly associated with time to BF before adjusting for clinicopathologic features (Table 3). After adjustment for clinicopathologic features, HMMR (p = 0.008), IGF1 (p = 0.015), SMAD4 (p = 0.016) remained significantly associated and new associations were observed for HOXC6 (p = 0.050) and MAP4K4 (p = 0.024). These data demonstrate that HMMR, HOXC6, IGF1, MAP4K4, and SMAD4 are associated with time to BF and that their associations are not fully explained by currently established clinicopathologic parameters.

**Table 3:** Biomarkers as predictors of time to biochemical failure using Cox regression models

	**N**	**Univariate**		Multivariate*	
**Variable**		**HR (95% CI)**	**P-value**	**HR (95% CI)**	**P-value**
ACPP	139	0.93 (0.63-1.39)	0.735	1.07 (0.59-1.93)	0.827
ADAM9	152	0.98 (0.63-1.54)	0.944	1.19 (0.75-1.91)	0.462
ALDH1A2	141	0.79 (0.47-1.32)	0.368	1.10 (0.62-1.95)	0.737
CASR	143	1.01 (0.65-1.57)	0.949	0.98 (0.58-1.66)	0.937
CCND1 (nuclear)	142	1.38 (1.01-1.88)	0.042	1.18 (0.87-1.61)	0.296
CCPG1	143	1.19 (0.76-1.88)	0.446	1.14 (0.69-1.86)	0.611
CD34 (avg vess area)	146	1.16 (0.74-1.83)	0.515	1.02 (0.60-1.74)	0.938
CD34 (avg vess per)	146	1.42 (0.91-2.21)	0.123	1.43 (0.84-2.43)	0.188
CD34 (avg lum area)	146	1.45 (0.95-2.22)	0.086	1.37 (0.83-2.27)	0.220
CD34 (avg vasc area)	146	1.02 (0.66-1.60)	0.915	0.89 (0.52-1.53)	0.682
CD34 (MVD)	146	1.11 (0.71-1.73)	0.652	0.97 (0.56-1.67)	0.901
CD34	146	1.11 (0.72-1.71)	0.648	1.04 (0.63-1.71)	0.881
CD44	148	1.07 (0.68-1.68)	0.764	1.06 (0.70-1.60)	0.786
CD44v6	143	1.23 (0.79-1.89)	0.360	1.35 (0.84-2.16)	0.217
CHGA	136	0.50 (0.24-1.04)	0.064	0.56 (0.26-1.21)	0.139
CHMP1A	146	1.31 (0.83-2.08)	0.244	1.35 (0.84-2.20)	0.218
EI24 (stroma)	140	1.24 (0.85-1.82)	0.265	1.36 (0.97-1.91)	0.077
EI24 (tumor)	140	1.32 (0.94-1.87)	0.114	1.32 (0.97-1.79)	0.077
ENO2	145	1.33 (0.92-1.93)	0.135	1.48 (0.92-2.38)	0.108
GADD45B	148	1.11 (0.72-1.73)	0.630	1.18 (0.78-1.77)	0.435
HA (stroma)	150	1.51 (0.99-2.29)	0.055	1.43 (0.95-2.17)	0.090
HAS2	148	1.27 (0.82-1.97)	0.287	1.47 (0.94-2.30)	0.090
HES6	140	0.80 (0.48-1.34)	0.401	0.69 (0.38-1.27)	0.238
HMMR	139	2.01 (1.23-3.30)	0.005	1.98 (1.20-3.29)	0.008
HOXC6	142	1.22 (0.79-1.89)	0.368	1.51 (1.00-2.28)	0.050
HYAL1	147	1.42 (0.92-2.21)	0.116	1.40 (0.87-2.25)	0.163
IGF1	147	1.57 (1.02-2.42)	0.039	1.67 (1.10-2.52)	0.015
IQCK	149	0.90 (0.58-1.40)	0.651	0.90 (0.59-1.38)	0.625
MAP4K4	145	1.44 (0.91-2.28)	0.121	1.76 (1.08-2.86)	0.024
MKI67 (nuclear)	142	1.43 (1.04-1.96)	0.026	1.29 (0.93-1.79)	0.125
PAGE4	145	0.68 (0.45-1.03)	0.069	0.88 (0.57-1.35)	0.566
PLIN2	115	1.21 (0.71-2.09)	0.481	1.07 (0.62-1.86)	0.801
PTEN	144	0.83 (0.54-1.27)	0.393	0.84 (0.51-1.38)	0.493
SIAH2 (nuclear)	144	1.46 (1.07-1.99)	0.016	1.37 (0.94-2.02)	0.104
SMAD4	130	1.76 (1.15-2.71)	0.010	1.73 (1.11-2.70)	0.016
SOX9	145	1.39 (0.90-2.16)	0.137	1.45 (0.93-2.27)	0.101
SPP1	128	1.07 (0.62-1.84)	0.805	0.76 (0.44-1.33)	0.338
SYP	133	0.88 (0.48-1.63)	0.681	0.95 (0.53-1.70)	0.869
TP53_Lab1 (nuclear)	148	1.23 (0.83-1.83)	0.305	1.19 (0.78-1.82)	0.431
TP53_Lab2 (nuclear)	143	0.97 (0.63-1.50)	0.906	0.93 (0.60-1.44)	0.741

MVD, microvessel density.

*Adjusted for age at prostatectomy, pre-op PSA, primary Gleason pattern, and Non-Localized Tumor Indicator.

### Multiple imputations and multibiomarker modeling of prostate cancer aggressiveness

Table 4 presents the frequency with which each biomarker appeared in the top 2, 3, 4 and 5 biomarkers across the ten imputed datasets. Three proteins (HMMR, SIAH2, and SMAD4) were consistently present in the top 2, 3, 4, and 5 most predictive biomarkers (4, 10, and 7 times in the top 3, respectively). A predictive model comprised of HMMR, SIAH2, and SMAD4 had an AUC of 0.69 (95% CI: 0.50, 0.78) for BF at 3 years and 0.70 (95% CI: 0.53, 0.87) for BF at 5 years (Table 5).

**Table 4:** Frequency of biomarkers appearing in the top 2, 3, 4, and 5 biomarkers with highest area under the ROC curve (AUC) across 10 imputed datasets

**Variable**	**Top 2**	**Top 3**	**Top 4**	**Top 5**
ACPP	0	0	0	0
ADAM9	0	0	0	0
ALDH1A2	0	0	0	0
CASR	0	0	0	0
CCND1 (nuclear)	0	0	0	0
CCPG1	0	0	0	0
CD34 (avg vess area)	0	0	0	0
CD34 (avg vess per)	0	1	4	5
CD34 (avg lum area)	0	1	3	3
CD34 (avg vasc area)	0	0	0	0
CD34 (MVD)	0	0	0	0
CD34	0	0	0	0
CD44	0	0	0	0
CD44v6	0	2	2	2
CHGA	0	1	1	2
CHMP1A	0	0	0	0
EI24 (stroma)	0	0	0	0
EI24 (tumor)	0	0	0	0
ENO2	0	0	1	1
GADD45B	0	0	0	0
HA (stroma)	1	1	1	3
HAS2	0	0	0	0
HES6	0	0	0	0
HMMR	2	4	6	6
HOXC6	0	0	0	0
HYAL1	0	0	0	0
IGF1	0	0	0	0
IQCK	0	0	0	0
MAP4K4	0	0	0	0
MKI67 (nuclear)	0	0	0	0
PAGE4	0	0	0	0
PLIN2	0	0	0	0
PTEN	0	0	0	0
SIAH2 (nuclear)	7	10	10	10
SMAD4	4	7	7	8
SOX9	0	0	0	0
SPP1	0	0	0	0
SYP	0	0	0	0
TP53_Lab1 (nuclear)	0	0	0	0
TP53_Lab2 (nuclear)	0	0	0	0

MVD, microvessel density.

**Table 5:** Area under the ROC curve (AUC) for 3-year and 5-year recurrence using a 3-biomarker model comprised of HMMR, SIAH2 (nuclear), and SMAD4

	**3-year recurrence**		**5-year recurrence**	
	**AUC (95% CI)**	**P-value**	**AUC (95% CI)**	**P-value**
Unadjusted AUC	0.708 (0.581-0.882)	0.004	0.721 (0.592-0.880)	0.002
Cross-validation adjusted AUC	0.685 (0.499-0.871)	0.052	0.702 (0.533-0.866)	0.024

## Discussion

While established clinicopathologic features including grade, stage, and preoperative PSA partially explain the variability in PCa outcome measured by BF after prostatectomy, further stratification of patients for more precise patient management may be possible with prognostic multibiomarker signatures. Many signatures and gene associations of PCa progression have been identified [6-18,23-25], but few markers emerge among multiple independent studies as potentially prognostic, outperform established clinicopathologic features, or become widely used. Identification of robust prognostic biomarkers will not only improve the clinical management of PCa, but is expected to provide an important bridge between pathology data and imaging data to facilitate development of novel imaging biomarkers which can be assessed noninvasively [22]. The present study focused on evaluating the prognostic utility of 33 proteins and investigating multibiomarker signatures that were predictive of BF. Although these proteins have been previously implicated in aggressive biologic behavior of PCa from diverse studies, to our knowledge this is the first study to examine these proteins in a single prostatectomy cohort using immunohistochemistry and objective automated digital pathology methods. We found that increased levels of several proteins in malignant epithelium were significantly associated with time to BF after adjustment for established clinicopathologic parameters including HMMR, HOXC6, IGF1, MAP4K4, and SMAD4. We also demonstrated that a 3-biomarker signature including HMMR, SMAD4, and SIAH2 was predictive of BF in our cohort, warranting further investigation of this signature in larger cohorts and imaging biomarker development studies.

HMMR is a multicompartmentalized receptor for the extracellular matrix carbohydrate HA with potentially oncogenic functions such as mediating cell motility and affecting mitotic spindle integrity [35]. Aberrant HA pathway signaling is implicated in prostate tumor cell proliferation, motility, angiogenesis, and metastasis [36]. Our results indicated that elevated HMMR in malignant epithelium was associated with BF which is similar to previous reports linking HMMR expression to the development of castration-resistant PCa and metastatic disease [12,37]. Further, low molecular weight HA fragments are catabolized from native HA by physical (reactive oxygen/nitrogen species) and enzymatic (hyaluronidase) mechanisms [38] and bind to HMMR to induce cell migration [39,40]. These data support the underlying model of aberrant HA-HMMR signaling in aggressive PCa and are encouraging for ongoing studies in our laboratories using small molecular inhibitors to disrupt fragmented HA-HMMR interactions for therapeutic targeting.

HOXC6 was identified in our cross-study analysis of gene expression datasets as part of an 11-gene signature of PCa aggressiveness. We further demonstrated an association between HOXC6 immunohistochemistry with time to BF in our PCa cohort. Similarly, Singh et al. identified HOXC6 in a 5-gene signature that was predictive of BF [15]. HOXC6 is an androgen-regulated gene which assists during development of normal tissues and progression of PCa [41]. Mouse models provide specific evidence for the interaction of HOXC6 with multiple downstream targets including bone morphogenic protein 7 (BMP7), fibroblast growth factor receptor 2 (FGFR2), and platelet-derived growth factor receptor α (PDGFRA) that promote PCa metastasis to the bone microenvironment [42]. Anti-apoptotic roles for HOXC6 have also been described in head and neck squamous cell carcinoma in which HOXC6 directly increases the promoter activity causing overexpression of Bcl-2 [43]. Similarly, HOXC6 represses the activity of pro-apoptotic genes neutral endopeptidase (NEP) and insulin-like growth factor binding protein 3 (IGFBP3) in PCa [41]. Although androgen receptor was not directly investigated in this study, we identified an association between elevated HOXC6 and BF in our cohort which along with these studies provides rationale for further investigation of HOXC6 in the PCa androgen receptor axis.

MAP4K4 is also a pro-migratory protein involved in mammalian development and increases tumor cell motility likely through c-Jun N-terminal kinase (JNK) [44]. Our findings indicated that increased MAP4K4 was significantly associated with time to BF. MAP4K4 is similarly incorporated in numerous independent gene expression signatures which are predictive of survival in colorectal cancer [45] and recurrence in prostate cancer [16,17]. Additionally, MAP4K4 is an independent prognostic factor for hepatocellular carcinoma and lung adenocarcinoma [46,47]. Xenograft tumor growth in mice using a hepatocellular cell line is substantially inhibited by RNA interference of MAP4K4 [46], indicating a potential therapeutic target that may be useful for treatment of PCa.

Higher protein levels of IGF1 were associated with time to BF in our PCa cohort. This observation was discordant from our initial cross-study analysis of independent gene expression datasets identifying downregulated IGF1 as part of an 11-gene signature of PCa aggressiveness (Additional file 12: Table S11). One recent study shows evidence for decreased IGF1 mRNA in local PCa compared to benign prostate, although IGF1 may still be involved in subsequent tumor progression as this study did not evaluate advanced or metastatic disease [48]. In contrast, our immunohistochemical findings correlate with most literature on the role of increased IGF1 in cancer progression. In androgen-independent PCa, IGF1 induces tumor cell motility by activation of αvβ3 integrin via the PI3-K/Akt pathway [49]. IGF1 signaling through PI3-K/Akt and β1 integrin similarly promotes adhesion and migration in multiple myeloma cells [50].

The PI3-K pathway is also upregulated in transgenic mice expressing tissue-specific IGF1 in prostate basal epithelial cells which leads to constitutive activation of IGF1R and increased development of PCa [51]. Importantly, meta-analysis of large datasets demonstrates an association between high serum IGF1 levels and moderately increased risk of PCa which may provide an important modifiable target in PCa patients [52].

In addition to IGF1, bioinformatic analysis identifies SMAD4 as a candidate gene likely to be a primary driver of PCa progression [53]. Reports of SMAD4 expression in prostate and other cancers are complex and variable, although mutation of the *SMAD4* gene appears rare during PCa progression [54]. In normal cells, SMAD4 is a critical component of the TGF-β signaling cascade and localizes to the nucleus after becoming activated to regulate TGF-β-responsive genes and inhibit cellular proliferation [55]. In this sense, SMAD4 may act as a tumor suppressor, which has been demonstrated by reduced expression of SMAD4 in PCa compared to BPH and normal prostate tissue [56]. Interestingly, in our cross-study analysis of gene expression datasets SMAD4 was present in the top 46 ranked genes (position 21) and inversely correlated with PCa aggressiveness (Additional file 4: Table S3). While Smad-dependent TGF-β signaling primarily functions to inhibit growth, the vast majority of tumors acquire resistance to these effects and tumor progression becomes stimulated by TGF-β in more advanced tumors [55]. Previous studies have linked increased SMAD4 to higher grade, stage, and DNA ploidy in PCa [57] and to infiltration of the myometrial wall in endometrioid endometrial cancer [58], which is consistent with our immunohistochemical analysis that elevated SMAD4 was significantly associated with time to BF. Similarly, SMAD4 shifts from its tumor suppressor role to an aggressiveness factor in a mouse model of breast cancer by promoting bone metastasis through TGF-β-activated expression of IL-11 [59]. Advanced primary prostate tumors often metastasize to the bone, potentially reflecting parallel events whereby bone-derived TGF-β offers an advantage to SMAD4-overexpressing prostate tumor cells and providing a possible therapeutic target in the TGF-β pathway.

HMMR, SMAD4, and SIAH2 comprised a multibiomarker signature that was predictive of BF in our cohort. SIAH2 is a RING finger ubiquitin ligase which controls the stability of multiple substrates, and under hypoxic conditions, causes ubiquitination/degradation of prolyl hydroxylase 3 and 1 [60]. This action increases the accumulation rate of HIF-1α and interacts with neuroendocrine-specific expression of FoxA2 leading to neuroendocrine PCa development and metastasis [14,60]. SIAH2 also contributes to castration-resistant PCa by targeting a subset of inactive androgen receptors for ubiquitination which increases the activity of androgen receptor target genes implicated in PCa progression [61]. Our results which identify SIAH2 in the multibiomarker signature predictive of BF are consistent with these studies and support a functional role for SIAH2 in contributing to aggressive subtypes of PCa.

This study and its conclusions are limited by a small sample size and short follow-up for some individuals. We collected data on 160 patients of which half were followed for less than 2.3 years. As a result, only 22 of the 160 patients in our sample experienced BF even though 67 patients had extraprostatic extension, lymph node involvement, or surgical margin involvement. It is likely that longer follow-up would result in more BF instances. The small number of BF’s observed in our sample is directly related to the relatively modest AUC (AUC ~0.7 for 3- and 5-year BF) observed for our 3-biomarker signature. A larger sample size and more events would provide more statistical power for developing a biomarker signature and more precision for evaluating the performance of the new biomarker signature. This retrospective hospital-based cohort study is additionally limited by lack of information regarding (neo)-adjuvant therapy. This is an important limitation of our study, since such treatments could potentially affect patient outcomes and thus our interpretations. We plan to complete a larger validation study to further refine our biomarker signature and to obtain more precise estimates of its performance as a classifier for BF.

## Conclusions

This study presents a unique methodology for evaluating the prognostic utility of PCa biomarkers. Because of the precious value of tissues represented in TMAs from large cohorts, we have tested 33 proteins by immunohistochemistry using this cohort of 160 patients in order to identify promising biomarkers for use in significantly larger validation studies [62]. Additionally, this study describes a 3-biomarker signature consisting of HMMR, SIAH2, and SMAD4 proteins which appears to be prognostic in our cohort. This signature may be useful in work currently underway to co-register detailed multibiomarker immunohistochemistry signature maps with multiparametric MR data, for the purpose of identifying MR biomarkers that assess PCa aggressiveness preoperatively *in vivo*[22].

## Competing interest

The authors declare that there are no conflicts of interest regarding the publication of this article.

## Authors’ contributions

AER, NKR, SCS, and GJM designed and coordinated the study. JSK and RIV provided statistical analysis. CLF performed immunohistochemical staining. AER, LOM, and JRT performed image and data analysis. JCH participated in study coordination and server administration. AER, NKR, GJM, JBM, EAT, CAW, and SCS helped to draft the manuscript. All authors read and approved the final manuscript.

## Pre-publication history

The pre-publication history for this paper can be accessed here:

http://www.biomedcentral.com/1471-2407/14/244/prepub

## Supplementary Material

Additional file 1 **Development of an *n*-gene signature of prostate cancer aggressiveness by cross-study examination of gene expression profiling data.** Click here for file

Additional file 2: Table S1 Fully normalized RNA expression data (Singh *et al.*) for the top 1000 most informative genes [500 genes with the most positive S_x_ (non-aggressive) and 500 genes with the most negative S_x_ (aggressive)] for 21 specimens in the Singh *et al.* dataset. Genes are ranked by decreasing informational content (|S_x_|). Click here for file

Additional file 3: Table S2 Fully normalized RNA expression data (Yu *et al.*) for the top 1000 most informative genes [500 genes with the most positive S_x_ (non-aggressive) and 500 genes with the most negative S_x_ (aggressive)] for 58 specimens in the Yu *et al.* dataset. Genes are ranked by decreasing informational content (|S_x_|). Click here for file

Additional file 4: Table S3 Top 46 genes (ranked by |weighted average S_x_|) with S_x_ values from each dataset. Expression values for genes with multiple probe set ID’s were averaged. S_x_ ratios were averaged and weighted to account for difference in sample size between datasets. Click here for file

Additional file 5: Table S4 Weighted voting calculations for the top 46 ranked genes using normalized gene expression data from the Singh *et al.* dataset (Additional file 2: Table S1) and weighted average S_x_ values (derived in Additional file 4: Table S3) using the voting equation: v = S_x_ [G_x_ - B_x_]. Click here for file

Additional file 6: Table S5 Weighted voting calculations for the top 46 ranked genes using normalized gene expression data from the Yu *et al.* dataset (Additional file 2: Table S1) and weighted average S_x_ values (derived in Table S3) using the voting equation: v = S_x_ [G_x_ - B_x_]. Click here for file

Additional file 7: Table S6 Aggressiveness predictions of *n*-gene models for each specimen in the Singh *et al.* dataset. These values result from the summation (*V*) of votes for genes added consecutively to the *n*-gene model: *V* = Σ_x_ v_x_, where the summation (*V*) is positive (non-aggressive; blue) or negative (aggressive; red). Statistical results for each *n*-gene model are displayed at the end of each row. Click here for file

Additional file 8: Table S7 Aggressiveness predictions of *n*-gene models for each specimen in the Yu *et al.* dataset. These values result from the summation (*V*) of votes for genes added consecutively to the *n*-gene model: *V* = Σ_x_ v_x_, where the summation (*V*) is positive (non-aggressive; blue) or negative (aggressive; red). Statistical results for each *n*-gene model are displayed at the end of each row. Click here for file

Additional file 9: Table S8 Fully normalized cDNA expression data (Lapointe *et al.*) for the ranked set of top 46 genes for the 28 specimens in the Lapointe *et al.* validation dataset. Click here for file

Additional file 10: Table S9 Weighted voting calculations for the top 46 ranked genes using normalized gene expression data from the Lapointe *et al.* validation dataset (Additional file 9: Table S8) and weighted average S_x_ values (derived in Table S3) using the voting equation: v = S_x_ [G_x_ - B_x_]. Click here for file

Additional file 11: Table S10 Aggressiveness predictions of *n*-gene models for each specimen in the Lapointe *et al.* validation dataset. These values result from the summation (*V*) of votes for genes added consecutively to the *n*-gene model: *V* = Σ_x_ v_x_, where the summation (*V*) is positive (non-aggressive; blue) or negative (aggressive; red). Statistical results for each *n*-gene model are displayed at the end of each row. Click here for file

Additional file 12: Table S11 Composition of the final 11-gene model validated in the Lapointe *et al.* dataset. Click here for file

Additional file 13: Table S12 Control tissues used in immunohistochemistry assays. Click here for file

Additional file 14: Figure S1 Representative images of selected control tissues demonstrating optimized immunohistochemistry. Click here for file

Additional file 15: Table S13 Reproducibility of assay methods given by N (%) patients for each combination of missing/observed for two independent runs and the correlation coefficients when data were observed in both runs. Click here for file
