# Supplementary material for: Evaluation of protein biomarkers of prostate cancer aggressiveness
Source: BMC Cancer. 2014 Apr 5;14:244. doi: 10.1186/1471-2407-14-244 (PMC4101830; doi:10.1186/1471-2407-14-244)
Supplement: Additional file 1 — Development of an n -gene signature of prostate cancer aggressiveness by cross-study examination of gene expression profiling data. [file 1471-2407-14-244-S1.pdf]

Additional file 1: Development of an *n*-gene signature of prostate cancer aggressiveness by cross-study examination of gene expression profiling data.

Datasets and processing

To investigate multigene signatures of prostate cancer (PCa) aggressiveness we evaluated gene expression profiling data from three independent PCa studies comparing tumor and normal prostate tissue. In our analysis we considered only primary tumor specimens from each study which had sufficient follow-up PSA data available.

A. Singh *et al* [1]: RNA microarray dataset containing 12,625 genes (Human Genome U95Av2 array; Affymetrix, Santa Clara, CA) was obtained from the Broad Institute database. Specimens included 21 primary prostate adenocarcinomas and were classified as aggressive (n=8; 38.1%) if the patient experienced two successive PSA values of 0.2 ng/mL or higher post-prostatectomy.

B. Yu *et al* [2]: RNA microarray dataset containing 12,625 genes (Human Genome U95A array; Affymetrix) was obtained from NCBI GEO DataSets (accession GSE6606). Specimens included 58 primary prostate adenocarcinomas and were classified as aggressive (n=26; 44.8%) if the patient experienced increased PSA value post-prostatectomy, local tumor invasion, and/or distant metastasis.

C. Lapointe *et al* [3]: cDNA microarray dataset containing 26,260 genes (custom cDNA spotted array; Stanford University) was obtained from NCBI GEO DataSets (accession GSE3933) and used as a validation dataset. Specimens included 28 primary prostate adenocarcinomas and were classified as aggressive (n=7; 25.0%) if the patient experienced >0.07 ng/mL rise in PSA post-prostatectomy and/or distant metastasis.

Raw RNA expression data were normalized within each dataset by applying the Robust Multichip Average (RMA) algorithm using Affymetrix Expression Console. A maximum threshold was set at the 99th percentile to mitigate the effects of extreme outliers. To correct for differences in differential intensity profiles between arrays within a dataset, arrays were least squares normalized by multiplying each array by 1/slope of an array constructed from the median expression value of each gene across all samples as described [4]. To provide a relative measure of fold-change which is more generalizable for cross-study analysis, arrays were adjusted by dividing each array was by its median expression value.

Raw cDNA expression data from Lapointe *et al* was not compatible with Affymetrix Expression Console software so our normalization technique for the Lapointe dataset was meant to approximate RMA for a two channel non-Affymetrix chipset using four steps: background correction, normalization, log correction, and linear modeling as described [5]. A procedure for the quantile normalization step is outlined by Bolstad *et al* [6]. The linear modeling step was omitted since cDNA expression data does not need probe set summarization normally required for RNA microarrays.

Gene filtering

Datasets from Singh *et al* [1] and Yu *et al* [2] were used as training datasets for building a supervised prediction model. To compare expression data from individual genes with tumor aggressiveness or non-aggressiveness we calculated the signal-to-noise ratio (S_x) for each gene: $S_x = (\mu_{NA} - \mu_A) / (\sigma_{NA} + \sigma_A)$ where, μ is the mean expression value and σ is the standard deviation of the expression values for a given gene *x* across all non-aggressive (NA) or aggressive (A) specimens in a single dataset as described [4, 7]. The 500 genes with the most positive S_x (“non-

aggressiveness genes") and 500 genes with the most negative S_x ("aggressiveness genes") were selected from each dataset to create a group of the 1000 most informative genes in each dataset. The top 1000 genes ranked by informational content (decreasing absolute value of S_x) in each training dataset are listed in Additional file 2: Table S1 (Singh *et al*) and Additional file 3: Table S2 (Yu *et al*). UniGene names were then mapped from Affymetrix probe set identifiers using GeneSifter (Geospiza, Seattle, WA). A total of 110 genes were shared between both top 1000 lists although complement component genes C2 and C7 were excluded (due to their ubiquitous presence in blood/tissues and unlikely specificity for PCa) resulting in 108 shared genes retained within each dataset for further analysis. Expression values for genes with multiple probe set identifiers were averaged. Signal-to-noise ratios for this selected gene set were averaged and weighted to account for difference in sample size between datasets. The list of genes was truncated at 50 as we aimed to validate these markers by immunohistochemistry; an additional 4 genes (UGT2B11, ITPR1, DNM2, and RFPL3) were subsequently excluded because they were not represented by cDNA probes in the Lapointe *et al* validation dataset. This final ordered list of 46 genes was ranked by decreasing informational content (absolute value of weighted average S_x) (Additional file 4: Table S3) and used in weighted voting analyses.

Supervised prediction using a weighted voting process

To examine the prognostic value of *n*-gene signatures derived from our ranked list of 46 genes, we utilized a weighted voting and class prediction process described by Ramaswamy *et al* [4] and originally developed by Golub *et al* [7]. Gene expression data and signal-to-noise ratios described above from Singh *et al* [1] and Yu *et al* [2] were used as inputs for training the

weighted voting algorithm to recognize aggressive from non-aggressive PCa. The independent cDNA expression dataset from Lapointe *et al* [3] was used for validation testing.

Aggressiveness or non-aggressiveness of a specimen *y* was predicted by the summation of weighted votes (*V*) from the *n* highest quality genes included in the model (consecutively added from the ranked set of top 46 genes in Additional file 4: Table S3). First, a vote (v_x) towards aggressive or non-aggressive was defined as $v_x = S_x\ (g_x^y - b_x)$ and assigned to each ranked gene based on the signal-to-noise quality factor (S_x) and proximity of a given specimen's gene expression level (g_x^y) to the gene's average expression level among non-aggressive (μ_{NA}) or aggressive specimens (μ_A) using its midpoint boundary ($b_x = (\mu_{NA} + \mu_A) / 2$). Weighted voting calculations are shown in Additional file 5: Table S4 (Singh *et al*) and Additional file 6: Table S5 (Yu *et al*). A final decision towards aggressiveness or non-aggressiveness was formulated by the summation of weighted votes (*V*) for *n* genes included in the model: $V = \Sigma_x\ v_x$ where the summation (*V*) is positive (non-aggressive) or negative (aggressive). Genes were consecutively added to the *n*-gene model by decreasing weighted average S_x beginning with the single highest quality gene from the ranked set of top 46 genes in Additional file 4: Table S3 (e.g.,1-gene model = CASR; 2-gene model = CASR+ACPP; 3-gene model = CASR+ACPP+GADD45B, etc.). Final *n*-gene voting predictions and statistical results for each model are shown in Additional file 7: Table S6 (Singh *et al*) and Additional file 8: Table S7 (Yu *et al*). Negative predictive values (NPV) and positive predictive values (PPV) were calculated under the assumption that a positive test result was defined by a prediction of aggressiveness and a negative test result was defined by a prediction of non-aggressiveness. Maximum NPVs occurred at 8 genes (100.0%; Singh *et al*) and 11 genes (72.7%; Yu *et al*).

Validation of n-gene models

Using the ranked set of top 46 genes (and weighted average S_x quality factors) generated from the Singh *et al* and Yu *et al* training datasets, we tested *n*-gene models using normalized cDNA expression data from a third independent validation dataset (Lapointe *et al* [3]) (Additional file 9: Table S8). Weighted voting was performed as described above using normalized cDNA gene expression data from the Lapointe *et al* validation dataset and weighted average S_x values derived from the training datasets (Additional file 10: Table S9). Final *n*-gene voting predictions and statistical results for each model using the Lapointe validation dataset are shown in Additional file 11: Table S10. Maximum NPV (94.7%) of a test for non-aggressiveness (optimized to identify aggressive tumors, even at the risk of scoring non-aggressive disease as "aggressive") occurred with an 11 gene model (Additional file 12: Table S11).

In the Singh *et al* dataset, this 11-gene model successfully identified 7 of 8 aggressive tumors and 10 of 11 non-aggressive tumors, resulting in 87.5% sensitivity and 90.1% specificity. In the Yu *et al* dataset, the 11-gene model successfully identified 17 of 24 aggressive tumors and 24 of 31 non-aggressive tumors, resulting in 70.8% sensitivity and 77.4% specificity. In the Lapointe *et al* dataset, the 11-gene model successfully identified 6 of 7 aggressive tumors and 15 of 16 non-aggressive tumors, resulting in 85.7% sensitivity and 93.8% specificity. Prediction of aggressiveness using the 11-gene model was significantly associated with actual prognosis in the Lapointe *et al* validation dataset using Fisher's Exact test (p=0.001).

References

1. Singh D, Febbo PG, Ross K, Jackson DG, Manola J, Ladd C, Tamayo P, Renshaw AA, D'Amico AV, Richie JP *et al*: **Gene expression correlates of clinical prostate cancer behavior**. *Cancer cell* 2002, **1**(2):203-209.

2. Yu YP, Landsittel D, Jing L, Nelson J, Ren B, Liu L, McDonald C, Thomas R, Dhir R, Finkelstein S *et al*: **Gene expression alterations in prostate cancer predicting tumor aggression and preceding development of malignancy**. *Journal of clinical oncology : official journal of the American Society of Clinical Oncology* 2004, **22**(14):2790-2799.
3. Lapointe J, Li C, Higgins JP, van de Rijn M, Bair E, Montgomery K, Ferrari M, Egevad L, Rayford W, Bergerheim U *et al*: **Gene expression profiling identifies clinically relevant subtypes of prostate cancer**. *Proc Natl Acad Sci U S A* 2004, **101**(3):811-816.
4. Ramaswamy S, Ross KN, Lander ES, Golub TR: **A molecular signature of metastasis in primary solid tumors**. *Nature genetics* 2003, **33**(1):49-54.
5. Irizarry RA, Hobbs B, Collin F, Beazer-Barclay YD, Antonellis KJ, Scherf U, Speed TP: **Exploration, normalization, and summaries of high density oligonucleotide array probe level data**. *Biostatistics* 2003, **4**(2):249-264.
6. Bolstad BM, Irizarry RA, Astrand M, Speed TP: **A comparison of normalization methods for high density oligonucleotide array data based on variance and bias**. *Bioinformatics* 2003, **19**(2):185-193.
7. Golub TR, Slonim DK, Tamayo P, Huard C, Gaasenbeek M, Mesirov JP, Coller H, Loh ML, Downing JR, Caligiuri MA *et al*: **Molecular classification of cancer: class discovery and class prediction by gene expression monitoring**. *Science* 1999, **286**(5439):531-537.
8. Haines AM, Larkin SE, Richardson AP, Stirling RW, Heyderman E: **A novel hybridoma antibody (PASE/4LJ) to human prostatic acid phosphatase suitable for immunohistochemistry**. *British journal of cancer* 1989, **60**(6):887-892.
9. Grutzmann R, Luttges J, Sipos B, Ammerpohl O, Dobrowolski F, Alldinger I, Kersting S, Ockert D, Koch R, Kalthoff H *et al*: **ADAM9 expression in pancreatic cancer is associated with tumour type and is a prognostic factor in ductal adenocarcinoma**. *British journal of cancer* 2004, **90**(5):1053-1058.
10. Sladek NE: **Human aldehyde dehydrogenases: potential pathological, pharmacological, and toxicological impact**. *Journal of biochemical and molecular toxicology* 2003, **17**(1):7-23.
11. Tfelt-Hansen J, Brown EM: **The calcium-sensing receptor in normal physiology and pathophysiology: a review**. *Critical reviews in clinical laboratory sciences* 2005, **42**(1):35-70.
12. Kapoor A, Satishchandra P, Ratnapriya R, Reddy R, Kadandale J, Shankar SK, Anand A: **An idiopathic epilepsy syndrome linked to 3q13.3-q21 and missense mutations in the extracellular calcium sensing receptor gene**. *Annals of neurology* 2008, **64**(2):158-167.
13. Donnellan R, Chetty R: **Cyclin D1 and human neoplasia**. *Molecular pathology : MP* 1998, **51**(1):1-7.
14. Pusztaszeri MP, Seelentag W, Bosman FT: **Immunohistochemical expression of endothelial markers CD31, CD34, von Willebrand factor, and Fli-1 in normal human tissues**. *The journal of histochemistry and cytochemistry : official journal of the Histochemistry Society* 2006, **54**(4):385-395.
15. Stauder R, Eisterer W, Thaler J, Gunthert U: **CD44 variant isoforms in non-Hodgkin's lymphoma: a new independent prognostic factor**. *Blood* 1995, **85**(10):2885-2899.
16. Horst E, Meijer CJ, Radaszkiewicz T, Ossekoppele GJ, Van Krieken JH, Pals ST: **Adhesion molecules in the prognosis of diffuse large-cell lymphoma: expression of a lymphocyte homing receptor (CD44), LFA-1 (CD11a/18), and ICAM-1 (CD54)**.

Leukemia : official journal of the Leukemia Society of America, Leukemia Research Fund, UK 1990, **4**(8):595-599.
17. Shtivelman E, Bishop JM: **Expression of CD44 is repressed in neuroblastoma cells**. *Molecular and cellular biology* 1991, **11**(11):5446-5453.
18. Kainz C, Kohlberger P, Tempfer C, Sliutz G, Gitsch G, Reinthaller A, Breitenecker G: **Prognostic value of CD44 splice variants in human stage III cervical cancer**. *European journal of cancer* 1995, **31A**(10):1706-1709.
19. Heider KH, Kuthan H, Stehle G, Munzert G: **CD44v6: a target for antibody-based cancer therapy**. *Cancer immunology, immunotherapy : CII* 2004, **53**(7):567-579.
20. Nobels FR, Kwekkeboom DJ, Bouillon R, Lamberts SW: **Chromogranin A: its clinical value as marker of neuroendocrine tumours**. *European journal of clinical investigation* 1998, **28**(6):431-440.
21. Portela-Gomes GM, Stridsberg M, Johansson H, Grimelius L: **Complex co-localization of chromogranins and neurohormones in the human gastrointestinal tract**. *The journal of histochemistry and cytochemistry : official journal of the Histochemistry Society* 1997, **45**(6):815-822.
22. Li J, Belogortseva N, Porter D, Park M: **Chmp1A functions as a novel tumor suppressor gene in human embryonic kidney and ductal pancreatic tumor cells**. *Cell cycle* 2008, **7**(18):2886-2893.
23. You Z, Xin Y, Liu Y, Sun J, Zhou G, Gao H, Xu P, Chen Y, Chen G, Zhang L *et al*: **Chmp1A acts as a tumor suppressor gene that inhibits proliferation of renal cell carcinoma**. *Cancer letters* 2012, **319**(2):190-196.
24. Zhao X, Ayer RE, Davis SL, Ames SJ, Florence B, Torchinsky C, Liou JS, Shen L, Spanjaard RA: **Apoptosis factor EI24/PIG8 is a novel endoplasmic reticulum-localized Bcl-2-binding protein which is associated with suppression of breast cancer invasiveness**. *Cancer research* 2005, **65**(6):2125-2129.
25. Lehar SM, Nacht M, Jacks T, Vater CA, Chittenden T, Guild BC: **Identification and cloning of EI24, a gene induced by p53 in etoposide-treated cells**. *Oncogene* 1996, **12**(6):1181-1187.
26. Seshi B, True L, Carter D, Rosai J: **Immunohistochemical characterization of a set of monoclonal antibodies to human neuron-specific enolase**. *The American journal of pathology* 1988, **131**(2):258-269.
27. Qiu W, David D, Zhou B, Chu PG, Zhang B, Wu M, Xiao J, Han T, Zhu Z, Wang T *et al*: **Down-regulation of growth arrest DNA damage-inducible gene 45beta expression is associated with human hepatocellular carcinoma**. *The American journal of pathology* 2003, **162**(6):1961-1974.
28. Wang L, Xiao X, Li D, Chi Y, Wei P, Wang Y, Ni S, Tan C, Zhou X, Du X: **Abnormal expression of GADD45B in human colorectal carcinoma**. *Journal of translational medicine* 2012, **10**:215.
29. Tammi RH, Tammi MI: **Chapter 19 - Hyaluronan in the Epidermis and Other Epithelial Tissues**. In: *Chemistry and Biology of Hyaluronan.* edn. Edited by Hari GG, Ph.D, D.Sc, Charles A. Hales MD. Oxford: Elsevier Science Ltd; 2004: 395-413.
30. Wang C, Tammi M, Guo H, Tammi R: **Hyaluronan distribution in the normal epithelium of esophagus, stomach, and colon and their cancers**. *The American journal of pathology* 1996, **148**(6):1861-1869.

31. Okuda H, Kobayashi A, Xia B, Watabe M, Pai SK, Hirota S, Xing F, Liu W, Pandey PR, Fukuda K *et al*: **Hyaluronan synthase HAS2 promotes tumor progression in bone by stimulating the interaction of breast cancer stem-like cells with macrophages and stromal cells**. *Cancer research* 2012, **72**(2):537-547.
32. Nykopp TK, Rilla K, Tammi MI, Tammi RH, Sironen R, Hamalainen K, Kosma VM, Heinonen S, Anttila M: **Hyaluronan synthases (HAS1-3) and hyaluronidases (HYAL1-2) in the accumulation of hyaluronan in endometrioid endometrial carcinoma**. *BMC cancer* 2010, **10**:512.
33. Haapa-Paananen S, Kiviluoto S, Waltari M, Puputti M, Mpindi JP, Kohonen P, Tynninen O, Haapasalo H, Joensuu H, Perala M *et al*: **HES6 gene is selectively overexpressed in glioma and represents an important transcriptional regulator of glioma proliferation**. *Oncogene* 2012, **31**(10):1299-1310.
34. Rein DT, Roehrig K, Schondorf T, Lazar A, Fleisch M, Niederacher D, Bender HG, Dall P: **Expression of the hyaluronan receptor RHAMM in endometrial carcinomas suggests a role in tumour progression and metastasis**. *Journal of cancer research and clinical oncology* 2003, **129**(3):161-164.
35. Wang C, Thor AD, Moore DH, 2nd, Zhao Y, Kerschmann R, Stern R, Watson PH, Turley EA: **The overexpression of RHAMM, a hyaluronan-binding protein that regulates ras signaling, correlates with overexpression of mitogen-activated protein kinase and is a significant parameter in breast cancer progression**. *Clinical cancer research : an official journal of the American Association for Cancer Research* 1998, **4**(3):567-576.
36. Bodey B, Bodey B, Jr., Siegel SE, Kaiser HE: **Immunocytochemical detection of the homeobox B3, B4, and C6 gene products in breast carcinomas**. *Anticancer research* 2000, **20**(5A):3281-3286.
37. Csoka AB, Scherer SW, Stern R: **Expression analysis of six paralogous human hyaluronidase genes clustered on chromosomes 3p21 and 7q31**. *Genomics* 1999, **60**(3):356-361.
38. Kasprzak A, Szaflarski W, Szmeja J, Andrzejewska M, Przybyszewska W, Kaczmarek E, Koczorowska M, Koscinski T, Zabel M, Drews M: **Differential expression of IGF-1 mRNA isoforms in colorectal carcinoma and normal colon tissue**. *International journal of oncology* 2013, **42**(1):305-316.
39. Maake C, Reinecke M: **Immunohistochemical localization of insulin-like growth factor 1 and 2 in the endocrine pancreas of rat, dog, and man, and their coexistence with classical islet hormones**. *Cell and tissue research* 1993, **273**(2):249-259.
40. Liu AW, Cai J, Zhao XL, Jiang TH, He TF, Fu HQ, Zhu MH, Zhang SH: **ShRNA-targeted MAP4K4 inhibits hepatocellular carcinoma growth**. *Clinical cancer research : an official journal of the American Association for Cancer Research* 2011, **17**(4):710-720.
41. Balka G, Ladinig A, Ritzmann M, Saalmuller A, Gerner W, Kaser T, Jakab C, Rusvai M, Weissenbock H: **Immunohistochemical characterization of type II pneumocyte proliferation after challenge with type i porcine reproductive and respiratory syndrome virus**. *Journal of comparative pathology* 2013, **149**(2-3):322-330.
42. Brinkmann U, Vasmatzis G, Lee B, Yerushalmi N, Essand M, Pastan I: **PAGE-1, an X chromosome-linked GAGE-like gene that is expressed in normal and neoplastic**

prostate, testis, and uterus. *Proceedings of the National Academy of Sciences of the United States of America* 1998, **95**(18):10757-10762.
43. Heid HW, Moll R, Schwetlick I, Rackwitz HR, Keenan TW: **Adipophilin is a specific marker of lipid accumulation in diverse cell types and diseases**. *Cell and tissue research* 1998, **294**(2):309-321.
44. Gimm O, Perren A, Weng LP, Marsh DJ, Yeh JJ, Ziebold U, Gil E, Hinze R, Delbridge L, Lees JA *et al*: **Differential nuclear and cytoplasmic expression of PTEN in normal thyroid tissue, and benign and malignant epithelial thyroid tumors**. *The American journal of pathology* 2000, **156**(5):1693-1700.
45. Zaman MS, Thamminana S, Shahryari V, Chiyomaru T, Deng G, Saini S, Majid S, Fukuhara S, Chang I, Arora S *et al*: **Inhibition of PTEN gene expression by oncogenic miR-23b-3p in renal cancer**. *PloS one* 2012, **7**(11):e50203.
46. Schmidt RL, Park CH, Ahmed AU, Gundelach JH, Reed NR, Cheng S, Knudsen BE, Tang AH: **Inhibition of RAS-mediated transformation and tumorigenesis by targeting the downstream E3 ubiquitin ligase seven in absentia homologue**. *Cancer research* 2007, **67**(24):11798-11810.
47. Wilentz RE, Su GH, Dai JL, Sparks AB, Argani P, Sohn TA, Yeo CJ, Kern SE, Hruban RH: **Immunohistochemical labeling for dpc4 mirrors genetic status in pancreatic adenocarcinomas : a new marker of DPC4 inactivation**. *The American journal of pathology* 2000, **156**(1):37-43.
48. Stuelten CH, Buck MB, Dippon J, Roberts AB, Fritz P, Knabbe C: **Smad4-expression is decreased in breast cancer tissues: a retrospective study**. *BMC cancer* 2006, **6**:25.
49. Morais da Silva S, Hacker A, Harley V, Goodfellow P, Swain A, Lovell-Badge R: **Sox9 expression during gonadal development implies a conserved role for the gene in testis differentiation in mammals and birds**. *Nature genetics* 1996, **14**(1):62-68.
50. Wang DH, Clemons NJ, Miyashita T, Dupuy AJ, Zhang W, Szczepny A, Corcoran-Schwartz IM, Wilburn DL, Montgomery EA, Wang JS *et al*: **Aberrant epithelial-mesenchymal Hedgehog signaling characterizes Barrett's metaplasia**. *Gastroenterology* 2010, **138**(5):1810-1822.
51. Brown LF, Berse B, Van de Water L, Papadopoulos-Sergiou A, Perruzzi CA, Manseau EJ, Dvorak HF, Senger DR: **Expression and distribution of osteopontin in human tissues: widespread association with luminal epithelial surfaces**. *Molecular biology of the cell* 1992, **3**(10):1169-1180.
52. Gould VE, Wiedenmann B, Lee I, Schwechheimer K, Dockhorn-Dworniczak B, Radosevich JA, Moll R, Franke WW: **Synaptophysin expression in neuroendocrine neoplasms as determined by immunocytochemistry**. *The American journal of pathology* 1987, **126**(2):243-257.
53. Baldi A, De Luca A, Esposito V, Campioni M, Spugnini EP, Citro G: **Tumor suppressors and cell-cycle proteins in lung cancer**. *Pathology research international* 2011, **2011**:605042.
54. Kaserer K, Schmaus J, Bethge U, Migschitz B, Fasching S, Walch A, Herbst F, Teleky B, Wrba F: **Staining patterns of p53 immunohistochemistry and their biological significance in colorectal cancer**. *The Journal of pathology* 2000, **190**(4):450-456.
