# Supplementary material for: Evaluation of protein biomarkers of prostate cancer aggressiveness
Source: BMC Cancer. 2014 Apr 5;14:244. doi: 10.1186/1471-2407-14-244 (PMC4101830; doi:10.1186/1471-2407-14-244)
Supplement: Additional file 12: Table S11 — Composition of the final 11-gene model validated in the Lapointe et al. dataset. [file 1471-2407-14-244-S12.pdf]

Additional file 12: Table S11. Composition of the final 11-gene model validated in the Lapointe dataset.

Rank	Gene	Description	Weighted Average S_x Score
1	CASR	calcium-sensing receptor	-0.41051
2	ACPP	acid phosphatase, prostate	0.38730
3	GADD45B	growth arrest and DNA-damage-inducible, beta	0.36906
4	PLIN2	perilipin 2	0.35674
5	ALDH1A2	aldehyde dehydrogenase 1 family, member A2	0.35287
6	ADAM9	ADAM metallopeptidase domain 9	0.34676
7	CCPG1	cell cycle progression 1	0.34670
8	HOXC6	homeobox C6	-0.34553
9	IQCK	IQ motif containing K	0.34255
10	IGF1	insulin-like growth factor 1 (somatomedin C)	0.34054
11	PAGE4	P antigen family, member 4 (prostate associated)	0.34008
