# Supplementary material for: Evaluation of protein biomarkers of prostate cancer aggressiveness
Source: BMC Cancer. 2014 Apr 5;14:244. doi: 10.1186/1471-2407-14-244 (PMC4101830; doi:10.1186/1471-2407-14-244)
Supplement: Additional file 13: Table S12 — Control tissues used in immunohistochemistry assays. [file 1471-2407-14-244-S13.pdf]

Additional file 13: Table S12. Control tissues used in immunohistochemistry assays.

Antibody	Localization	Control Tissue
ACPP	Cytoplasmic	Pancreas (islet cells) [9]; kidney (loops of Henle) [9]
ADAM9	Cytoplasmic; frequent luminal positivity	Pancreas (islet cells) [10]
ALDH1A2	Cytoplasmic	Testis (seminiferous tubules) [11]; kidney (EC) [11]; liver (EC) [11]
CASR	Cytoplasmic; frequent membrane and luminal positivity	Parathyroid (EC) [12]; kidney (EC) [12]; cerebral cortex (NC) [13]
CCND1	Nuclear	Mantle cell lymphoma [14]; breast cancer [14]
CCPG1[a]	Cytoplasmic; membranous	*Testis (sertoli cells); cerebral cortex (NC)*
CD34	Cytoplasmic	Kidney (glomeruli and interstitial capillaries) [15]; lung (alveolar capillaries) [15]
CD44	Membranous	Tonsil (lymphoid follicle mantle cells) [16]; DLBCL [17]; neuroblastoma (negative) [18]
CD44v6	Membranous	Cervix (squamous epithelium) [19]; cervical cancer [19]; breast (myoepithelium) [20]; breast cancer [20]
CHGA	Cytoplasmic; occasional perinuclear	Paraganglioma [21]; colon (endocrine cells) [22]
CHMP1A	Cytoplasmic; nuclear	Pancreas (ductal cells) [23]; kidney [24]
EI24	Cytoplasmic; occasional membranous	Breast (myoepithelium) [25]; pancreas (islet cells) [26]
ENO2	Cytoplasmic	Endometrium (nerve fibers) [27]
GADD45B	Cytoplasmic; frequent apical accentuation	Liver (hepatocytes) [28]; colon (decreased) [29]
HA (bHABP)	Interstitial streaming/cytoplasmic; membranous; pericellular	Skin (basal and lower spinous squamous cells) [30]; colon (lamina propria) [31]
HAS2	Cytoplasmic; frequent apical accentuation	Breast cancer [32]; breast (decreased) [32]; endometrial cancer [33]; endometrium (decreased) [33]
HES6	Nuclear; weak cytoplasmic	Glioblastoma [34]
HMMR	Cytoplasmic; nuclear; membranous	Endometrial cancer [35]; endometrium (decreased) [35]; breast cancer [36]; breast (decreased) [36]
HOXC6	Cytoplasmic; nuclear	Breast cancer [37]
HYAL1	Cytoplasmic; nuclear	Kidney (EC and proximal tubules) [38]; spleen (endothelial cells) [38]
IGF1	Cytoplasmic; frequent luminal positivity	Colon (EC) [39]; pancreas [40]
IQCK[b]	Cytoplasmic; frequent apical accentuation	*Stomach (EC); adrenal cortex*
MAP4K4	Cytoplasmic; frequent apical accentuation	Liver (hepatocytes and endothelial cells) [41]
MKI67	Nuclear	Lung (proliferating cells) [42]
PAGE4	Cytoplasmic; frequent supranuclear accentuation	Endometrium (secretory cells) [43]; placenta (syncytiotrophoblasts) [43]
PLIN2	Cytoplasmic; lipid droplets	Liver (hepatocytes) [44]; adrenal cortex [44]
PTEN	Cytoplasmic; nuclear	Thyroid (EC) [45]; kidney [46]
SIAH2	Nuclear	Colon cancer [47]; colon (decreased) [47]
SMAD4	Cytoplasmic; nuclear	Pancreas (islet, acinar, and ductal cells) [48]; breast (EC) [49]
SOX9	Cytoplasmic; nuclear	Testis (sertoli cells) [50]; stomach (pit-gland transition zone) [51]
SPP1	Cytoplasmic	Kidney (distal tubules) [52]; endometrium (secretory cells) [52]
SYP	Cytoplasmic; membranous	Colon cancer [53]; paraganglioma [53]
TP53	Nuclear	Lung cancer [54]
	Nuclear	Colon cancer [55]

bHABP, biotinylated hyaluronan binding protein; DLBCL, diffuse large B-cell lymphoma; EC, epithelial cells; NC, neuronal cells

[a]CCPG1 is a novel protein lacking published tissue controls. Tissues described here are representative observations from our own tissue analyses.

[b]IQCK is a novel protein lacking published tissue controls. Tissues described here are representative observations from our own tissue analyses.
