# Supplementary figures and images for: Evaluation of protein biomarkers of prostate cancer aggressiveness
Source: BMC Cancer. 2014 Apr 5;14:244. doi: 10.1186/1471-2407-14-244 (PMC4101830; doi:10.1186/1471-2407-14-244)

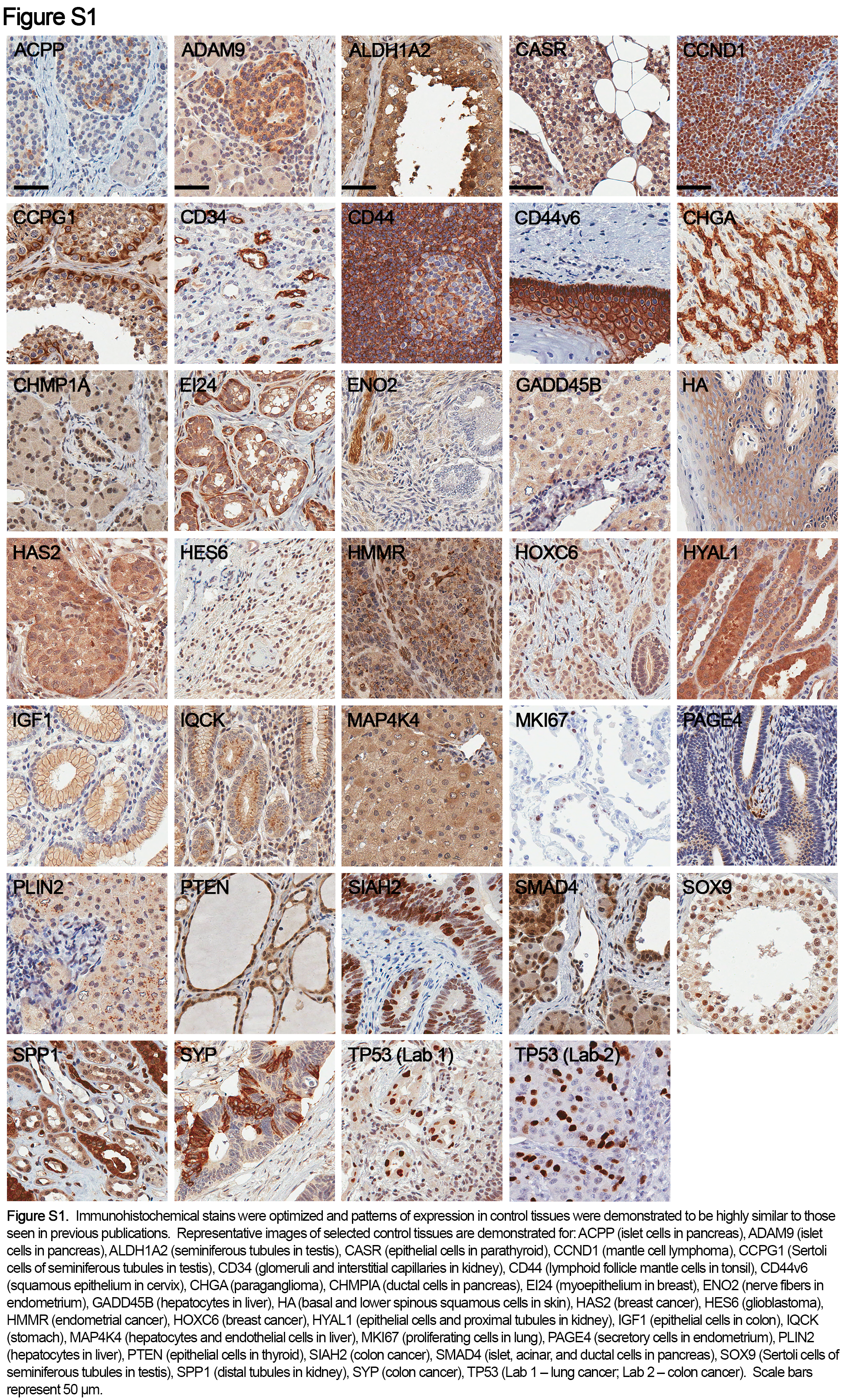

Supplement: Additional file 14: Figure S1 — Representative images of selected control tissues demonstrating optimized immunohistochemistry. [file 1471-2407-14-244-S14.tiff]
