# Supplementary material for: Evaluation of protein biomarkers of prostate cancer aggressiveness
Source: BMC Cancer. 2014 Apr 5;14:244. doi: 10.1186/1471-2407-14-244 (PMC4101830; doi:10.1186/1471-2407-14-244)
Supplement: Additional file 15: Table S13 — Reproducibility of assay methods given by N (%) patients for each combination of missing/observed for two independent runs and the correlation coefficients when data were observed in both runs. [file 1471-2407-14-244-S15.pdf]

Additional file 14: Table S13. Reproducibility of assay methods given by N (%) patients for each combination of missing/observed for two independent runs and the correlation coefficients when data were observed in both runs.

Variable	Both Observed	Run 1 Observed Run 2 Missing	Run 1 Missing Run 2 Observed	Both Missing	Correlation (95% CI)
CCND1	136 (80.0%)	6 (3.5%)	12 (7.1%)	16 (9.4%)	0.81 (0.66, 0.90)
CD44	135 (79.4%)	13 (7.6%)	7 (4.1%)	15 (8.8%)	0.62 (0.50, 0.72)
CD44v6	141 (82.9%)	2 (1.2%)	11 (6.5%)	16 (9.4%)	0.72 (0.62, 0.82)
HA	138 (81.2%)	12 (7.1%)	5 (2.9%)	15 (8.8%)	0.69 (0.59, 0.79)
HAS2	134 (78.8%)	14 (8.2%)	3 (1.8%)	19 (11.2%)	0.57 (0.44, 0.67)
HMMR	121 (71.2%)	18 (10.6%)	13 (7.6%)	18 (10.6%)	0.54 (0.44, 0.64)
HYAL1	144 (84.7%)	3 (1.8%)	6 (3.5%)	17 (10.0%)	0.58 (0.46, 0.68)
SMAD4	129 (75.9%)	1 (0.6%)	20 (11.8%)	20 (11.8%)	0.57 (0.43, 0.69)
